# Malignant Degeneration of Burn Scars (Marjolin's Ulcer): A Retrospective Case Series of Nine Patients

**DOI:** 10.7759/cureus.106624

**Published:** 2026-04-07

**Authors:** Ismail Hailouma, Soufyane Elkadiri, Mohamed Amine Gazanayi, Doha Arreyouchi, Ayat Allah Oufkir

**Affiliations:** 1 Plastic Surgery, Mohammed VI University Hospital, Oujda, MAR; 2 Plastic and Reconstructive Surgery, Mohammed VI University Hospital, Oujda, MAR

**Keywords:** burn scar, marjolin's ulcer, reconstructive surgery, sarcoma, squamous cell carcinoma, verrucous carcinoma

## Abstract

Introduction

Malignant degeneration of burn scars, commonly referred to as Marjolin's ulcer, is a rare but aggressive complication of chronic scars. Squamous cell carcinoma is the most frequent histological type, although other variants may occur. The aim of this study was to describe the epidemiological, clinical, histopathological, and therapeutic characteristics of patients treated in our department.

Methods

We conducted a retrospective descriptive study including patients with histologically confirmed malignant tumors arising on burn scars, treated between January 2019 and March 2026 at Mohammed VI University Hospital in Oujda, Morocco. Demographic, clinical, histological, therapeutic, and outcome data were analyzed.

Results

Nine patients were included, with a mean age of 58.6 years (range 47-71 years). There was a clear male predominance (88.9%). The lower extremities were the most commonly affected site (55.6%). Histologically, eight patients presented tumors within the squamous cell carcinoma spectrum, including three verrucous variants, while one patient developed a high-grade sarcomatous transformation on the same burn scar. Treatment was primarily surgical, including wide excision, reconstruction with skin grafts or flaps, and amputation in advanced cases. The amputation rate was 22.2%, and adjuvant radiotherapy was administered in 44.4% of cases.

Conclusion

Marjolin's ulcer remains a rare but aggressive entity requiring early diagnosis and prompt surgical management. Any chronic or unstable burn scar showing recent changes should be biopsied. The possibility of rare histological transformations highlights the need for careful pathological evaluation.

## Introduction

Marjolin's ulcer refers to a malignant transformation developing in a chronic scar or old wound, particularly in a burn scar. Although rare, this condition is recognized for its aggressiveness, with a greater risk of local invasion, recurrence, and lymph node involvement than primary cutaneous carcinomas [1,2]. Reviews of the literature report an incidence ranging from approximately 0.7% to 2% in burn scars, a marked predominance of squamous cell carcinoma, and a mean latency period that frequently exceeds 20 years [1,3-7].

In their review of 412 neoplasms arising in burn scars, Kowal-Vern and Criswell reported that 71% were squamous cell carcinomas, 12% basal cell carcinomas, 6% melanomas, and 5% sarcomas, the mean age at diagnosis was 50 years, and the mean latency period was 31 years [1]. Kerr-Valentic et al. further emphasized the rarity of Marjolin's ulcer while highlighting its substantial clinical aggressiveness [2]. More recently, Kanth et al. analyzed 1,016 patients in a systematic review and confirmed that surgery remains the mainstay of treatment [8].

The aim of the present study was to analyze the epidemiological, clinical, histopathological, therapeutic, and prognostic characteristics of patients treated in our department, with particular emphasis on histological diversity and the importance of early diagnosis.

## Materials and methods

This retrospective descriptive study was conducted in the Department of Burn, Plastic, Reconstructive, and Aesthetic Surgery at Mohammed VI University Hospital in Oujda, Morocco, between January 2019 and March 2026.

Eligible cases were identified through a retrospective review of departmental hospitalization records, operative logs, and pathology reports for patients with histologically confirmed malignant tumors arising on burn scars. Cases without pathological confirmation of malignancy and chronic non-neoplastic ulcerations were excluded. Duplicate records were carefully reviewed and removed to avoid double-counting.

One patient presented two successive histological diagnoses on the same burn scar, namely, recurrent squamous cell carcinoma followed by a high-grade malignant sarcomatous proliferation. This patient was counted only once in the demographic analysis, while the disease course was detailed in the Results and Discussion sections.

Data were extracted from the medical records by the authors using a standardized review process covering clinical, operative, pathological, and follow-up data. The variables analyzed included age, sex, burn etiology, anatomical location, latency period when available, clinical presentation, histological type, locoregional or deep extension, treatment, reconstruction method, adjuvant therapy, and outcome.

Statistical analysis was performed using Microsoft Excel (Microsoft Corporation, Redmond, Washington, United States). Continuous variables were expressed as mean values with ranges, while categorical variables were presented as frequencies and percentages. Given the purely descriptive nature and the small sample size of the series, no inferential statistical analysis was performed.

This study was conducted in accordance with institutional ethical standards. According to local regulations, formal institutional review board approval was not required for retrospective studies using anonymized patient data.

## Results

Nine patients were included. The mean age was 58.6 years (range 47-71 years). Eight patients were male, and one was female, indicating a marked male predominance. The lower extremities were the most commonly involved site (5/9, 55.6%), followed by the upper extremities (3/9, 33.3%) and the trunk (1/9, 11.1%).

**Figure 1:**
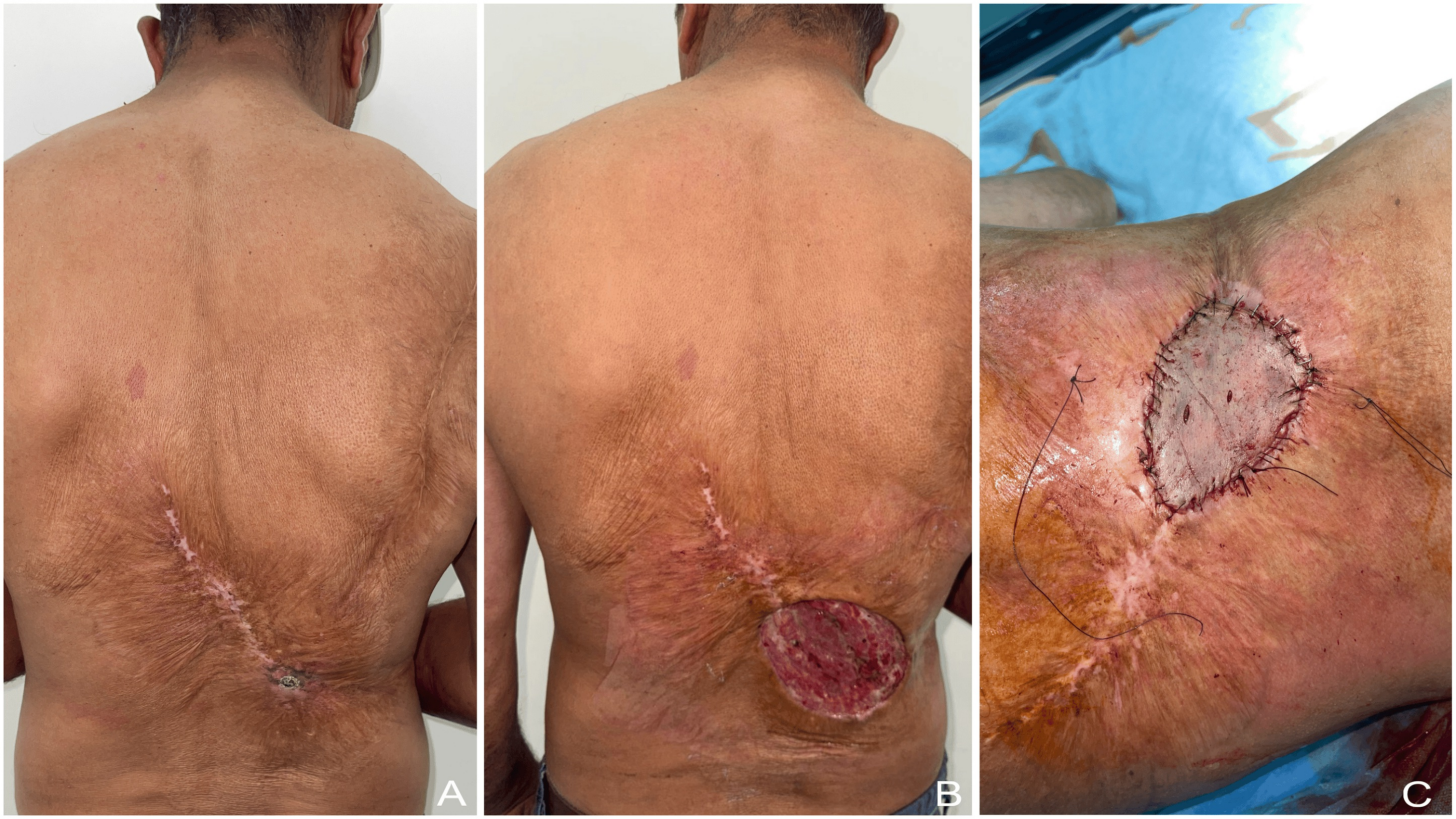
Malignant degeneration arising from a chronic burn scar of the back (A) Ulcerated lesion developing on a long-standing burn scar. (B) Defect after wide surgical excision. (C) Reconstruction using a split-thickness skin graft.

Clinical presentation was dominated by chronic non-healing ulceration, ulcerative or exophytic tumor growth, friability, bleeding on contact, foul odor, and progressive local tissue destruction. Figure 1 illustrates a representative lesion arising on a chronic burn scar of the back, together with the post-excisional defect and reconstruction using a split-thickness skin graft.

**Figure 2:**
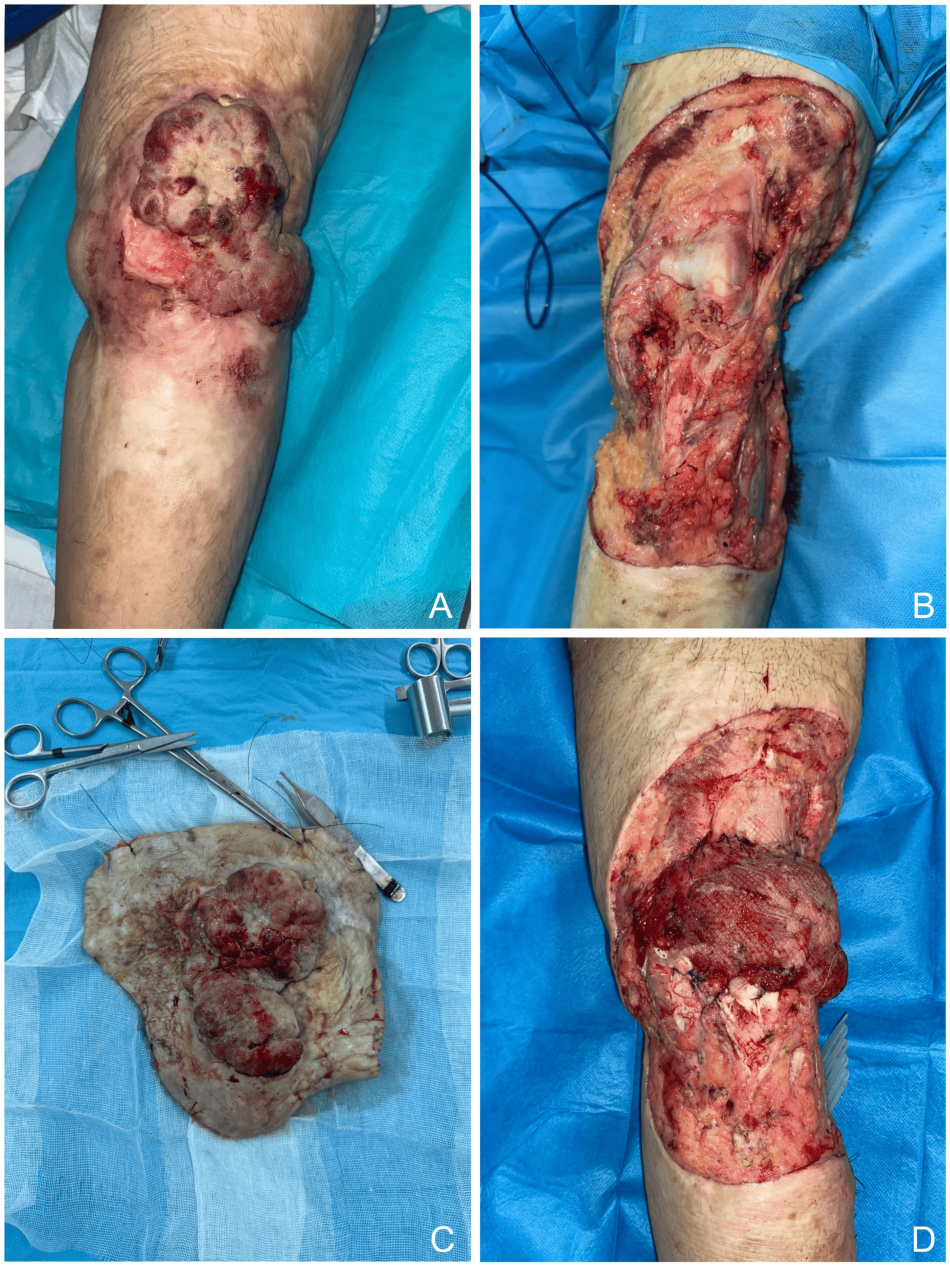
Surgical management of malignant degeneration on a burn scar of the knee (A) Ulcerative and exophytic tumor on a chronic burn scar. (B) Wide local excision of the lesion. (C) Surgical specimen after resection. (D) Reconstruction of the defect using a lateral gastrocnemius muscle flap.

Figure 2 shows a locally advanced knee lesion managed by wide excision, with the resulting defect, surgical specimen, and reconstruction using a lateral gastrocnemius muscle flap.

**Figure 3:**
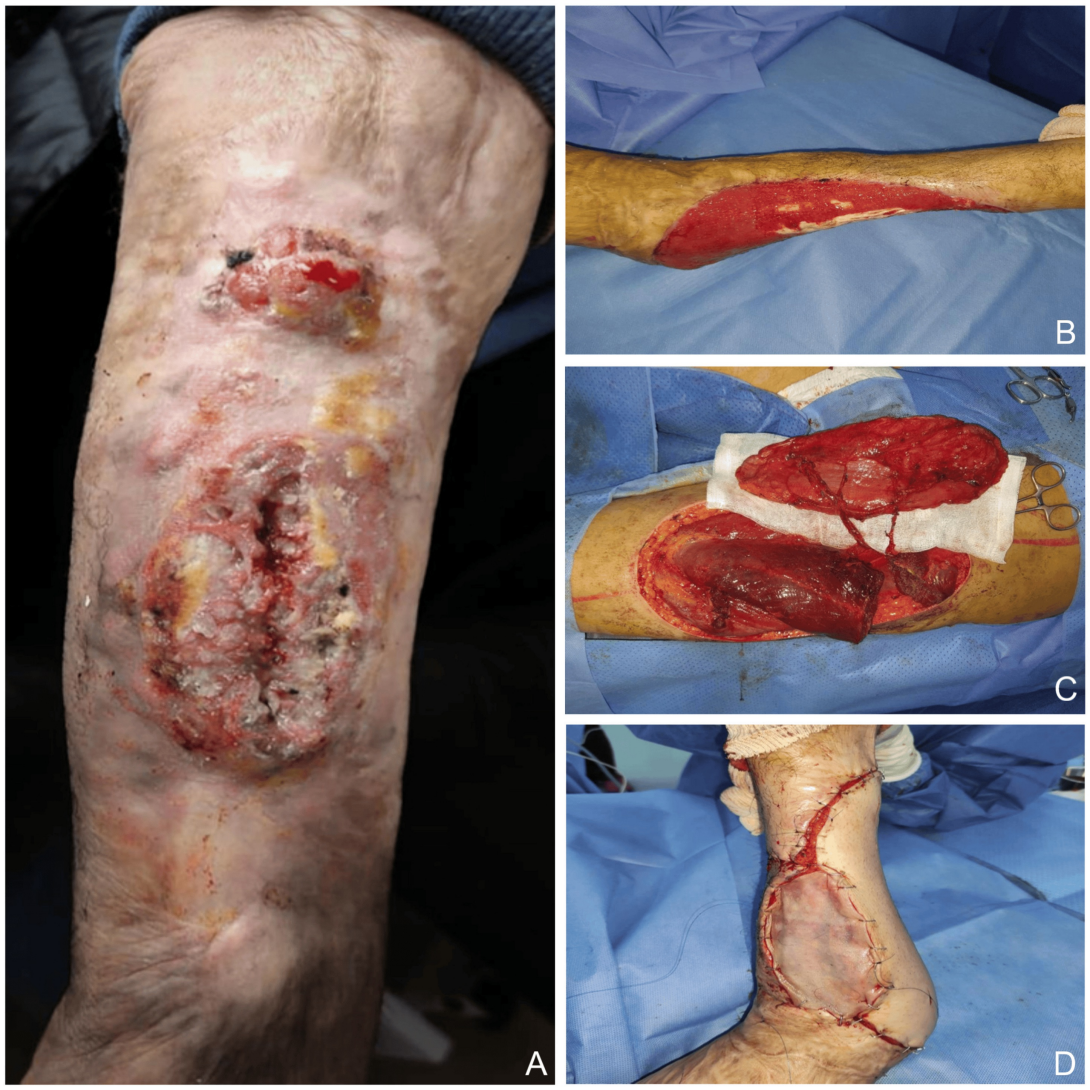
Advanced malignant degeneration on a burn scar of the leg with surgical excision and reconstruction (A) Chronic ulcerative and infiltrative lesion arising on a burn scar of the leg. (B) Post-excisional defect following wide local resection. (C) Harvesting of an anterolateral thigh flap. (D) Reconstruction using the anterolateral thigh flap combined with split-thickness skin grafting.

Figure 3 illustrates an advanced lower limb lesion requiring wide local resection followed by reconstruction using an anterolateral thigh flap combined with split-thickness skin grafting.

Histologically, eight patients (88.9%) had tumors within the squamous cell carcinoma spectrum, including five conventional squamous cell carcinomas and three verrucous variants (33.3%). One patient (11.1%) developed a high-grade malignant sarcomatous proliferation on the same burn scar of the knee after an initial course marked by recurrent squamous cell carcinoma.

The disease was frequently diagnosed at an advanced stage, as reflected by the extent of local lesions, the depth of surgical excision required, and the need for complex reconstruction or ablative procedures in selected cases. These findings are summarized in Tables 1-3.

**Table 1:** Clinical and histopathological characteristics of the nine patients

Patient	Age	Sex	Burn etiology	Location	Latency	Histology	Key findings
1	56	Male	Flame burn	Left leg	23 years	Well-differentiated invasive squamous cell carcinoma	Inguinal lymphadenopathy, locoregional extension
2	71	Female	Scald burn	Right leg	10 years	Well-differentiated invasive squamous cell carcinoma	Locally advanced lesion
3	64	Male	Flame burn	Right popliteal fossa	30 years	Well-differentiated verrucous squamous cell carcinoma	Suspicious pulmonary nodules
4	51	Male	Petroleum burn	Right forearm	11 years	Invasive keratinizing squamous cell carcinoma	Ulnar bone lysis, lymphadenopathy
5	47	Male	Burn scar (knee)	Left knee	24 years	Recurrent squamous cell carcinoma followed by high-grade sarcomatous transformation	Recurrence, immunohistochemistry revision, amputation indicated
6	59	Male	Lightning burn	Back	38 years	Micro-invasive verrucous squamous cell carcinoma	Complete excision
7	67	Male	Flame burn	Hand/wrist	29 years	Confirmed squamous cell carcinoma	Complete excision
8	56	Male	Electrical burn	Left forearm	35 years	Verrucous squamous cell carcinoma	Exophytic keratotic lesion
9	56	Male	Flame burn	Leg	23 years	Squamous cell carcinoma with deep invasion	Lymph node dissection, radiotherapy

**Table 2:** Treatment modalities and outcomes

Patient	Main treatment	Reconstruction/additional procedure	Adjuvant therapy	Outcome
1	Transfemoral amputation	Inguinal lymph node dissection	Radiotherapy	Suspected recurrence, lost to follow-up
2	Wide local excision	Split-thickness skin graft	Radiotherapy	No recurrence reported
3	Wide local excision	Split-thickness skin graft	No adjuvant therapy administered	Follow-up continued at another institution
4	Wide local excision	Axillary/epitrochlear dissection and free anterolateral thigh flap	Radiotherapy	Favorable outcome
5	Repeated excisions and then amputation indicated	Gastrocnemius flap	No adjuvant therapy administered	Aggressive course
6	Wide local excision	Split-thickness skin graft	No adjuvant therapy administered	Favorable outcome
7	Wide local excision	Split-thickness skin graft	No adjuvant therapy administered	Favorable outcome
8	Wide local excision	Split-thickness skin graft	No adjuvant therapy administered	Limited follow-up
9	Wide excision/radical surgery	Split-thickness skin graft	Radiotherapy	Poor outcome

**Table 3:** Summary of the series

Variable	Result
Total number of patients	9
Mean age	58.6 years
Male patients	88.9% (8/9)
Lower limb involvement	55.6% (5/9)
Upper limb involvement	33.3% (3/9)
Trunk involvement	11.1% (1/9)
Squamous cell carcinoma spectrum	88.9% (8/9)
Verrucous variants	33.3% (3/9)
Secondary sarcomatous transformation	11.1% (1/9)
Amputation rate	22.2% (2/9)
Adjuvant radiotherapy	44.4% (4/9)

## Discussion

Our series confirms the classic characteristics of malignant degeneration of burn scars, including a predominance of male patients, a prolonged latency period, an ulcerative-exudative presentation, and a high frequency of lesions affecting the lower extremities. These findings are consistent with previously published studies by Kowal-Vern and Criswell, Kerr-Valentic et al., and Xiang et al., all of which report a clear male predominance and latency periods frequently exceeding 20-30 years [1,2,9]. Xiang et al., in a series of 140 cases, reported a mean age of 53.3 years and a mean latency of 28.8 years [9].

In our study, the mean age of 58.6 years falls within the range reported in large series. This distribution reflects the delayed onset of malignant transformation, which is widely attributed to prolonged chronic inflammation and persistent scar instability [1,5]. The male predominance observed in our series (88.9%) is also consistent with the literature and is likely multifactorial, reflecting increased exposure to severe burns in occupational and domestic environments [2,6].

The latency period remains a hallmark of Marjolin's ulcers. Conventional data place this interval between 20 and 40 years [1,3,4]. In our series, latency ranged from 10 to 38 years, with a mean latency of 24.8 years. These findings are consistent with previous reports, although shorter latency periods have also been described, particularly in unstable scars or areas subjected to chronic mechanical stress [3-6].

The pathophysiological mechanisms underlying malignant transformation are thought to involve chronic inflammation, repeated microtrauma, poor vascularization, impaired lymphatic drainage, and reduced local immune surveillance within scar tissue [4-7]. The chronicity of the scar environment appears to play a central role in carcinogenesis. This is supported in our series by several patients who were initially managed by secondary intention healing without early definitive skin coverage.

Clinically, our patients presented with a highly characteristic pattern, including chronic ulceration, ulcerated or nodular lesions, bleeding on contact, pain, exudation, and occasionally foul odor. These features are widely recognized as suggestive of malignant transformation [4-6]. Any recent change in a longstanding scar, particularly the appearance of a proliferative lesion, induration, fissuring, or non-healing ulceration, should prompt immediate biopsy [4,5].

Imaging studies played a crucial role in the assessment of advanced cases, particularly for evaluating deep or bone invasion. In our series, bone involvement was identified in two patients. Xiang et al. reported bone invasion in up to 32.9% of cases [9]. In our experience, magnetic resonance imaging (MRI) and computed tomography (CT) imaging were essential in guiding surgical decision-making, particularly when amputation was considered or complex reconstruction was planned.

The histological spectrum in our series is dominated by squamous cell carcinoma, as expected. Copcu et al. reported a similar predominance in a series of 31 cases [3]. More recent reviews by Saaiq and Ashraf, Bazaliński et al., and Khan et al. and the systematic review by Kanth et al. confirm that squamous cell carcinoma accounts for more than 80% of Marjolin's ulcers [5-8]. In our series, five patients had conventional squamous cell carcinoma, and three had verrucous variants. The presence of verrucous forms highlights the histological heterogeneity of these tumors and underscores the importance of careful pathological evaluation [5,6].

One of the most noteworthy findings in our study is the occurrence of a high-grade sarcomatous transformation arising on a background of recurrent squamous cell carcinoma. Although rare, such transformations have been reported. Kowal-Vern and Criswell estimated that sarcomas account for approximately 5% of malignancies arising in burn scars [1]. Additional reports have described mesenchymal tumors, including dermatofibrosarcoma protuberans, developing on chronic burn scars [10,11].

This observation emphasizes the need for thorough pathological reassessment in cases of atypical evolution, recurrence, or discordant clinical behavior. Expert histological review, immunohistochemistry, and, when necessary, molecular analyses are essential to ensure diagnostic accuracy. From an academic perspective, the coexistence of epithelial and mesenchymal malignancies within the same series represents a distinctive and valuable contribution.

Surgical management remains the cornerstone of treatment [4,8]. The primary objectives are complete oncological excision with histologically clear margins and reconstruction adapted to the size, depth, and location of the defect. Skin grafts are particularly useful for facilitating postoperative surveillance, whereas flap-based reconstruction is indicated when critical structures are exposed [4,8]. The systematic review by Kanth et al. confirms that wide excision is the most commonly employed strategy, with reconstruction tailored on a case-by-case basis [8].

Our series highlights a broad spectrum of surgical management strategies, including split-thickness skin grafts, lateral gastrocnemius flap, free anterolateral thigh flap, and radical procedures in advanced cases. Two patients required amputation, representing 22.2% of the series. This rate is consistent with previous reports. Bozkurt et al., in a series of 16 cases, showed that amputation may remain necessary in locally advanced disease despite standardized management protocols [4]. Indications include bone involvement, neurovascular invasion, inability to obtain adequate surgical margins, or failure of previous treatment.

In our series, lymph node involvement and the use of adjuvant radiotherapy (44.4%) were observed mainly in the most advanced cases. The literature consistently shows that Marjolin's ulcers have a higher metastatic potential than primary cutaneous squamous cell carcinomas [1,2]. This underlines the importance of systematic staging and multidisciplinary management, particularly in large, deep, or clinically aggressive tumors.

Prevention remains a critical aspect of management. Early coverage of deep burns, long-term surveillance of unstable scars, and prompt biopsy of suspicious lesions are universally recommended [1,5-7]. Kowal-Vern and Criswell emphasized the preventive value of early excision and grafting in deep burns [1]. This point is especially relevant in our setting, where several patients were initially managed by secondary healing.

These findings further support the need for early diagnosis and individualized surgical management in order to improve outcomes and reduce morbidity.

The main limitations of our study include its retrospective design, the small sample size inherent to a single-center case series, the heterogeneity of certain cases, and the limited level of detail available for some patients. In addition, the descriptive design does not allow analytical inference or broad generalization of the findings. Nevertheless, this series retains substantial clinical value because of its histological diversity, the inclusion of advanced presentations, and the rarity of sarcomatous transformation arising on a burn scar.

A comparative analysis of our findings with major published series is presented in Table 4.

**Table 4:** Comparison with major published series

Study	Year	Number of cases	Predominant histology	Mean latency	Key findings
Kowal-Vern and Criswell [1]	2005	412	Squamous cell carcinoma (71%)	31 years	Sarcomas (5%)
Copcu et al. [3]	2003	31	Squamous cell carcinoma	19 years	Distal limb predominance
Bozkurt et al. [4]	2010	16	Squamous cell carcinoma (87.5%)	>30 years	Standardized surgical protocol
Xiang et al. [9]	2019	140	Squamous cell carcinoma	28.8 years	Bone invasion (32.9%)
Kanth et al. [8]	2021	1016	Squamous cell carcinoma (94%)	Not reported	Systematic review
Our study	2026	9	Squamous cell carcinoma (88.9%) + verrucous variants (33.3%) + 1 sarcoma (11.1%)	24.8 years	Amputation (22.2%)

## Conclusions

Malignant degeneration of burn scars remains a rare but severe condition. Squamous cell carcinoma is the most common histological form, but the occurrence of secondary sarcomatous transformation underscores that the histological spectrum may be broader than expected. Any old burn scar that becomes unstable, ulcerated, proliferative, painful, malodorous, or prone to bleeding should undergo prompt biopsy. Surgical resection remains the cornerstone of treatment, combined with appropriate reconstruction and, in advanced cases, ablative procedures.
